# The GABA_A_ receptor modulator zolpidem augments hippocampal-prefrontal coupling during non-REM sleep

**DOI:** 10.1038/s41386-022-01355-9

**Published:** 2022-06-18

**Authors:** Flavie Kersanté, Ross J. Purple, Matthew W. Jones

**Affiliations:** School of Physiology, Pharmacology and Neuroscience, University of Bristol, Biomedical Sciences Building, University Walk, Bristol, BS8 1TD UK

**Keywords:** Neuroscience, Non-REM sleep

## Abstract

Benzodiazepines and ‘Z-drugs’ (including zolpidem and zopiclone) are GABA_A_ receptor (GABA_A_R) positive modulators commonly prescribed as hypnotics to treat insomnia and/or anxiety. However, alongside sedation, augmenting GABA_A_R function may also alter coordinated neuronal activity during sleep, thereby influencing sleep-dependent processes including memory consolidation. We used simultaneous recordings of neural population activity from the medial prelimbic cortex (PrL) and CA1 of the dorsal hippocampus (dCA1) of naturally sleeping rats to detail the effects of zolpidem on network activity during the cardinal oscillations of non-REM sleep. For comparison, we also characterized the effects of diazepam and 4,5,6,7-tetrahydroisoxazolo(5,4-c)pyridin-3-ol (THIP/gaboxadol), which acts predominantly at extra-synaptic GABA_A_Rs. Zolpidem and THIP significantly increased the amplitudes of slow-waves, which were attenuated by diazepam. Zolpidem increased hippocampal ripple density whereas diazepam decreased both ripple density and intrinsic frequency. While none of the drugs affected thalamocortical spindles in isolation, zolpidem augmented the temporal coordination between slow-waves and spindles. At the cellular level, analyses of spiking activity from 523 PrL and 579 dCA1 neurons revealed that zolpidem significantly enhanced synchronized pauses in cortical firing during slow-wave down states, while increasing correlated activity within and between dCA1 and PrL populations. Of the drugs compared here, zolpidem was unique in augmenting coordinated activity within and between hippocampus and neocortex during non-REM sleep. Zolpidem’s enhancement of hippocampal-prefrontal coupling may reflect the cellular basis of its potential to modulate offline memory processing.

## Introduction

Over the past 50 years, hypnotics including benzodiazepines (BZ; e.g. diazepam, temazepam) and Z-drugs (e.g. zolpidem, zopiclone) have been used to treat anxiety and insomnia [1, 2]. Hypnotics do produce modest, clinically-relevant reductions in sleep latency [3], but at the expense of side-effects, dependency, and risk of abuse. Consequently, cognitive behavioural therapy for insomnia (CBT-I) is increasingly recommended as a first-line treatment [4, 5]. However, some recent studies suggest that combining CBT-I with managed, short-term zolpidem can increase the rate of symptom improvement [6, 7]; but see [4]. The effectiveness of such combination therapy may reflect the potential of Z-drugs to enhance memory consolidation [8–11]—and hence the efficacy of CBT—through actions on sleep-dependent neural network activity.

Non-rapid eye movement sleep (NREM) is characterized by the occurrence of: slow-wave activity (SWA, 0.5–4 Hz) predominantly reflecting periodic changes in membrane potential and spiking of neocortical cells; spindles (10–15 Hz) oscillating in thalamo-cortical loops, and fast ‘ripple’ oscillations (125–200 Hz) in CA1 of the hippocampus [12–15]. The features and neural circuitry of these hallmark oscillations are highly conserved in mammals, making them useful translational biomarkers of brain function and drug action. Rather than acting individually, the temporal coordination of NREM oscillations critically reflects their functions in aligning experience-dependent limbic-cortical activity [16–18]: ripples are temporally correlated with spindles [19, 20] and spindle-ripple synchrony is orchestrated by slow-waves [21–23]. Consequent coordination of distributed neural ensembles spanning thalamus, hippocampus, and neocortex is thought to be essential for sleep-dependent memory consolidation, hence deep-brain, optogenetic or transcranial stimulation can be used to enhance hippocampal-cortical and/or slow-wave-spindle coupling and augment memory consolidation in rodents or humans [24–28].

Sleep disruption, the core feature of primary insomnia and a common feature of several psychiatric and neurodegenerative disorders, is often associated with aberrant NREM oscillations [29–31] which may, in turn, contribute to or exacerbate memory impairments. For example, SWA can be reduced in depression [32, 33], spindles and their coupling with slow-waves (SW) are consistently compromised in schizophrenia [34, 35] and impaired coupling between SW and spindles is associated with Alzheimer’s Disease pathology in both mouse models [36] and elderly humans [37]. Drugs able to modulate NREM neurophysiology, therefore, remain of considerable interest, though their ability to sustain limbic-cortical interactions appears key to memory consolidation. Thus, despite some beneficial effects on sleep, the GABA reuptake inhibitor tiagabine disrupts SW-spindle coupling and does not augment memory in healthy participants [38], and the GABA-A receptor (GABA_A_R) modulator eszopiclone fails to rescue SW-spindle coupling, hence memory impairment, in schizophrenia patients [39].

BZ and Z-drugs show differential effects on sleep and oscillatory activity, presumably reflecting their different sites of action. BZ are non-specific positive allosteric modulators of GABA_A_Rs with a predominant hypnotic effect on α1 subunits in the mammalian central nervous system [40]. While BZ treatment can prolong sleep time, it may worsen sleep quality by increasing stage 2 NREM sleep at the expense of deeper stages of sleep. Specifically, BZ drugs have been shown to enhance sleep spindle activity but decrease SWA in humans [41, 42]. BZ also decrease SWA in rodents [43–46], in addition to reducing hippocampal ripple activity [47, 48].

Z-drugs are also positive modulators of GABA_A_Rs, with high selectivity for α1 subunits [1]. The reported effects of Z-drugs on oscillatory activity are less consistent. Zolpidem has been shown to either increase, decrease, or have no effect on SWA and spindles in humans [49–51] and rodents [52, 53]. Zolpidem has also been reported to either enhance [48] or reduce ripple power [47]. In addition to BZ and Z-drugs, other sedative compounds which act as agonists of extrasynaptic δGABA_A_Rs (which are highly expressed in the thalamus) have also been shown to influence oscillatory activity [54]. THIP is one such GABAARs agonist which has been shown to enhance SWA whilst decreasing spindle activity in both humans and rodents [50, 54]. The effect of THIP on ripple activity has not previously been reported.

Whilst these previous studies have provided insights into the effects of GABA_A_R modulators on individual features of sleep neurophysiology at the network level, it remains critical to understand how these drugs might affect underlying cellular activity and the interactions between coordinated oscillations spanning cortical and subcortical structures. Deciphering the cellular and network effects of these different drugs on cortico-limbic activity would inform their use in promoting sleep and associated memory processing [8, 9, 17]. Here, we therefore detail the acute effects of zolpidem, diazepam, and THIP on NREM oscillations and corresponding neuronal activity in the prefrontal cortex and hippocampus of naturally sleeping rats. In particular, we test the hypothesis that zolpidem’s potential to enhance sleep-dependent memory consolidation may reflect its effects on coordinated limbic-cortical activity during sleep.

## Materials and methods

### Drugs

Zolpidem (Tocris Bioscience, UK) was dissolved in 10% Ethanol, 20% polyethylene glycol, and 70% distilled water at a final concentration of 3 mg/ml. Diazepam (Tocris Bioscience, UK) was dissolved in 10% dimethyl sulfoxide, 20% Cremophor EL and 70% saline at a final concentration of 4 mg/ml. THIP hydrochloride (Tocris Bioscience, UK) was dissolved in saline at a final concentration of 4 mg/ml. Each drug and their respective vehicles were intraperitoneally (i.p.) administered at a volume of 1 ml/kg in counterbalanced order across rats. Control experiments were undertaken with both saline and each drug’s respective vehicle; neither saline nor vehicles had any significant effects on neural activity, so only saline experiments are shown here.

Doses were optimized on the basis of previous studies: 3 mg/kg zolpidem has been shown to increase sleep duration and SWA in rats [55] and reaches peak plasma concentration within 15 min, with a half-life of approximately 20 min [56]; 4 mg/kg diazepam caused sedation as well as a decrease in SWA in rodents [57–59], again achieving peak concentration within 10 min of injection and clearing with a half-life in the order of 1 h [60]; 4 mg/kg of THIP has been shown to increase NREM sleep and enhance SWA in rats [61].

### Animals and surgery

All procedures were performed in accordance with the UK Animals Scientific Procedures Act (1986) and were approved by the University of Bristol Animal Welfare and Ethical Review Board. Rats were housed individually in transparent, high-roofed cages to avoid damage to cranial implants; cages were enriched with wooden blocks, cardboard tubes, and flowerpots. Rooms were temperature- and humidity-controlled, with lights on at 08:00 and off at 20:00.

Five adult male Lister Hooded rats (350–400 g, 12–16 weeks of age, Charles River UK) were each implanted with 20 extracellular tetrode recording electrodes: 10 into deep layers of the right prelimbic cortex (PrL, +3.2 mm, −0.6 mm from bregma) and 10 into CA1 in the right dorsal hippocampus (−3.6 mm, +2.2 mm from bregma) under isoflurane recovery anaesthesia. Tetrodes were connected to an electrode interface board (EIB 36, Neuralynx, MT). Stainless steel screws were placed in the skull overlying the cerebellum and connected to the ground on the EIB via silver wires. An i.p injection of the analgesic buprenorphine (0.05 mg/kg) was given post-surgery. During the 15–21 days following surgery, the independently moveable tetrodes were lowered into the brain, targeting the prelimbic subdivision of the prefrontal cortex (3–4 mm ventral to brain surface) and pyramidal cell layer in the dorsal CA1 (verified by the characteristic burst mode of single-unit firing and the presence of large-amplitude sharp-wave ripple events in the LFP signal).

### Electrophysiological recording protocol

Before surgery and during tetrode adjustment, animals were habituated to a sound-attenuating recording chamber. Behaviour was constantly monitored via four video cameras. Single unit activity and local field potentials (LFP) were recorded simultaneously using Digital Lynx hardware and Cheetah software (Neuralynx, MT) using local references, which were placed in the superficial prefrontal cortex and in the white matter overlying the hippocampus. LFPs were sampled at 2 kHz and filtered between 0.1–475 Hz; extracellular action potentials were sampled at 32 kHz and filtered between 0.6–6 kHz.

**Fig. 1:**
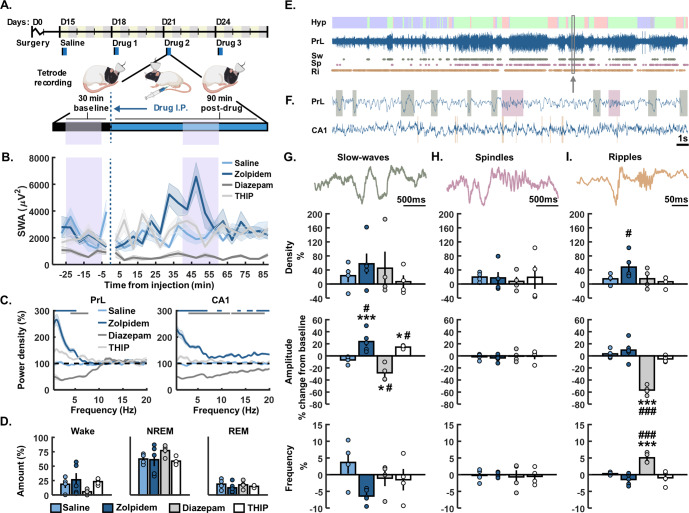
Effects of zolpidem, diazepam, and THIP on non-REM sleep oscillations. **A** Experimental design. After recovery from tetrode implantation, local field potentials and single unit activity were recorded for 30 min after sleep onset, 5 min were allowed for drug administration, and then a further 90 min were recorded post-injection. A 2–3 day wash out period was used between drugs under a randomised design. **B** Average slow wave activity (SWA) across the full recording period for each of the drugs (mean ± SEM). Shaded areas denote the 5–25-min baseline and 40–60 min post injection periods that were used for all analyses. Dotted line marks the injection time. **C** Average power density in the prelimbic cortex (PrL, left) and hippocampal CA1 (right) during the 20 min post-injection analysis period, as a percentage of the 20 min pre-injection baseline. Lines at the top of each plot show frequencies that were significantly different between saline and zolpidem (blue) or diazepam (grey). No differences were found for THIP. **D** Average amount of wake (left), NREM sleep (middle), and REM sleep (right) during the 20-min post-injection analysis period. No differences were found in the amount of sleep or wake between any of the drugs. **E** Representative example from one animal showing a hypnogram (Hyp) across the whole 90-min post-injection recording period. Blue = wake, green = NREM sleep, red = REM sleep. The corresponding raw LFP signal from the prelimbic cortex is shown below as well as rasters of all detected slow waves (Sw), spindles (Sp), and ripples (Ri). **F** Zoomed in 30 s period of NREM sleep (denoted by the arrow in **E**.) showing raw LFP from the PrL and CA1 with shaded areas showing detected slow waves (green), spindles (pink), and ripples (orange). Slow-waves/spindles were recorded and detected from the PrL and ripples from the dCA1. **G** Slow-wave, **H** spindle, and **I** ripple features. Top shows one example of each oscillation during NREM sleep (raw data, depolarisation upward deflections). Below shows average density, amplitude, and frequency for each oscillation after each drug administration. No significant differences were found for event duration (not shown). The results are expressed in percentage change compared to baseline. Asterisks denote statistical significance in comparison to saline injection: *p < 0.05 and ***p < 0.001. Hashes represent statistical differences to baseline: #, *p* < 0.05 and ###, *p* < 0.001., one-way ANOVA followed by Bonferroni post-hoc test. 4 rats were used for saline injection, 4 for diazepam, 5 for zolpidem and 4 for THIP. Error bars denote S.E.M across rats. Circles show data from individual rats.

Recordings were performed between 14:00 and 19:00, with drug injections at approximately 15:00, 5 h prior to onset of the rats’ wake phase (lights off). On each experimental day, each rat was placed in the recording chamber and baseline recording started once the animal was asleep (based on visual scoring). After 30 min baseline recording, the rat received an i.p injection of either zolpidem (3 mg/kg), diazepam (4 mg/kg), THIP (4 mg/kg), or saline (1 ml/kg); all 5 rats received zolpidem, whereas diazepam, THIP, and saline were tested in 4 rats. Injections occurred within a 5 min window and briefly awoke the rat; recording was then continued for the following 90 min. Each drug was administered successively in an order randomised across rats (Fig. 1A), with experimenter blinded to drug condition. The half-lives of these drugs in rat range from 20 min–1 h, so 2–3 days between injections allowed for complete clearance [56, 60, 62]. At the end of experimental procedures, rats were deeply anaesthetised with sodium pentobarbital and electrolytic lesions were performed at each electrode site. Rats were then transcardially perfused with 4% paraformaldehyde and 50 µm brain sections were stained with thionine to verify the electrodes placement (Fig. 3A/B).

### Analysis of vigilance state

Wake, REM or NREM arousal states were scored manually in 4 s windows (with the Open-source visbrain “Sleep” software [63]) using CA1 and PrL LFP (high theta/SWA ratio during Wake and REM, low during NREM), EMG (high power during wake, very low during REM, intermediate during NREM), and extracted movement from video. Epochs were scored as artefacts if at least one of the two LFP signals was contaminated by signs of eating, drinking, or gross movements. Sleep onset latency was calculated as the time at which the first epoch of NREM sleep was scored.

### Analysis of local field potentials

All analyses beyond Fig. 1B were restricted to 20 min windows, corresponding to 5–25 min pre-injection baseline and 40–60 min post-injection (shaded in Fig. 1B). All data were processed in Matlab (Mathworks, MA). Multitaper Fourier analyses (Chronux toolbox [64];) were used to calculate LFP power spectra and individual LFP oscillatory events (slow-waves, spindles, and ripples) were detected using custom MATLAB routines ([65] available here: https://gitlab.com/ubartsch/sleepwalker). In brief, LFP traces were first band pass filtered (slow-waves: 0.5–4 Hz, spindles: 8–16 Hz, ripples: 125–220 Hz) and then transformed giving a z-score. Slow-waves were directly detected from the filtered z-score signal with a threshold of 3.5 times the SD of the whole signal in both negative and positive direction. Slow-wave amplitude was measured between the initial positive peak marking onset and the maximal negative trough, which corresponded to the hyperpolarised down-state in our recordings. Only waves that contained a negativity larger than 50 µV were included. Slow-wave ‘intrinsic frequency’ was defined as the reciprocal of the wave period, i.e. 1/(end time–start time). For detection of sleep spindles and hippocampal ripples, the z-score of the filtered signal was rectified and determined using a cubic spline interpolation between the maxima of the rectified signal. This envelope was then used for detection of threshold crossing. Spindles were detected using a threshold of 3.5 SD of the signal; start and finish times of the spindle were also calculated (using a threshold 1.5 SD of the signal). Spindles were rejected if they were shorter than 350 ms or longer than 4 s; if two spindle events were separated by less than 0.5 s, they were merged into one. Ripples were detected using a threshold of 3.5 SD (2 SD for start and end times) and rejected if they were shorter than 50 ms or longer than 500 ms. If gaps of less than 50 ms occurred between ripples, they were treated as one event. Slow-wave, spindle, and ripple detection was performed for the baseline and post-drug recordings independently. To account for local LFP events and intermediate state transitions, we did not restrict our detections to NREM sleep. However, 95 ± 1.2% (mean ± SEM) of detected slow-waves, 85 ± 3.8% of detected spindles, and 68 ± 4.3% of detected ripples occurred during NREM sleep.

Power density was calculated using the MATLAB pwelch function with 4 s windows, no overlap, 0.25 Hz frequency bins. SWA across time was calculated by averaging the 4 s power density between 0.5–4 Hz and then averaging across 5 min bins. Phase-amplitude coupling (PAC) was quantified by calculating the Modulation Index, which relates the higher-frequency amplitude envelope of one (modulated) signal to the lower-frequency, instantaneous phase of another (the modulator); MATLAB code is available from [66].

### Analysis of single units

Single units were isolated off-line using automated clustering software (KlustaKwik 1.7; K. Harris) followed by verification and manual refinement using MClust 3.5 (A.D. Redish). Inclusion criteria were set to isolation distance 15.0 for PrL cells and isolation distance 10.0 for CA1 cells. Putative pyramidal cells were classified based on their waveforms (spike width >200 μs) and mean firing rate (less than 5 Hz).

Only units with mean firing rates of >0.15 Hz during the 120 min of recording were included in the analysis. The bursting index was defined as the proportion of interspike intervals (ISI) less than 20 ms. Peri-event histograms were used to analyze changes in unit firing rates during ripples and negative-going slow-wave oscillations. For this, firing rates were calculated around the peak of the oscillation (slow-waves: ±500 ms around peak, 5 ms bins; ripples: ±250 ms around oscillations, 2 ms bins) and values were normalized by conversion to z-score. Statistical analysis was then performed in the interval ±40 ms for ripples and ±250 ms for slow-waves (corresponding to the average length of ripples and slow-waves, respectively). To avoid spurious correlations based on low activity units, only units firing more than 200 spikes within the 20 min baseline and 20 min post-drug injection windows (firing rate = 0.16 Hz) were included in the cross-correlation analyses. Cross-correlations were calculated with 2 ms bin size and normalized by conversion to z-score. Statistical analysis was performed using the mean cross-correlation around the peak of cross-correlation (±100 ms for CA1 and PrL and 0-200 ms for PrL-CA1).

**Fig. 2:**
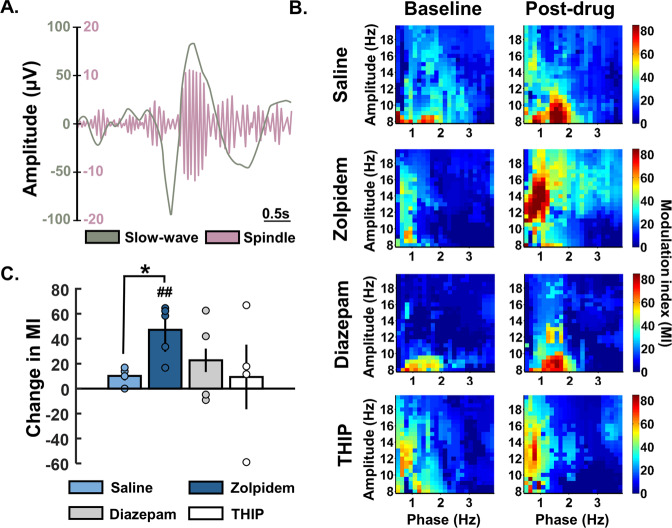
Phase amplitude coupling (PAC). **A** Schematic of a detected spindle during non-REM sleep filtered between 12–15 Hz and the same signal filtered between 0.5–4 Hz revealing underlying slow-wave activity. **B** PAC between 0.5–4 Hz and 8–20 Hz pre and post drug administration based on the modulation index (MI). **C** To specifically compare PAC within slow-wave and spindle frequency range, the maximal MI between 0.5–1.5 Hz (phase) and 10–16 Hz (amplitude) before and after drug injection were extracted for each animal and the change in PAC (Mipost – Mipre) was compared. Circles show data from individual rats. Asterisks denote statistical significance in comparison to saline injection: *p < 0.05, two sample *t*-test with unequal variance. Hashes represent statistical differences to baseline: ##, *p* < 0.01, one sample *t*-test. Error bars denote S.E.M across rats.

All bar graphs represent means ± S.E.M (across animals) and boxplots show mean, median, 25^th^, and 75^th^ percentiles (across units). Changes in comparison to baseline were obtained by subtracting the baseline values from the post-injection values for each drug and saline or as percentage of baseline. Normality of the different distributions were tested using the Shapiro–Wilks test and equal variances were tested using a Bartlett test. Depending on the distribution, comparison between saline and drugs were assessed either by a one-way ANOVA (AOV) followed by a Bonferroni pairwise comparison test (BC) or a Kruskal–Wallis (KW) test followed by the Dunns-Sivac (DS) post-hoc test. The phase-amplitude coupling analysis (Fig. 2) was an exception to this since the data were not of equal variance and one-sample or two-sample *t*-tests with unequal variance were used instead.

## Results

### Zolpidem augments NREM oscillations and their coupling

Each of the three GABA_A_ receptor modulators were first assessed for their effects on 0.5–4 Hz slow-wave activity. Zolpidem led to the largest increase in SWA reaching its peak during the 20 min post-injection analysis window, whereas diazepam led to a continued suppression of SWA throughout the recording period (Fig. 1B). However, none of the drugs showed a significant difference in comparison to saline for any of the time points after controlling for multiple testing (KW: *p* > 0.05). The remainder of our analyses were restricted to 5–25 min pre-injection and 40–60 min post-injection to adequately capture comparable pharmacokinetic-pharmacodynamic windows across all drugs.

In the prelimbic cortex (PrL), average power density during these 20-min windows revealed a significant increase in power around 0.5–5 Hz from pre- to post-injection of zolpidem compared to saline (Fig. 1C; significance for each 0.25 Hz bin, analysis of variance AOV: *p* < 0.05; followed by Bonferroni pairwise comparisons BC: *p* < 0.05). In contrast, diazepam induced a broad decrease in power <10 Hz, with a significant decrease around 4–8 Hz (AOV: *p* < 0.05, BC: *p* < 0.05). In CA1, zolpidem increased, and diazepam decreased power more broadly across frequencies (0.5–20 Hz) in comparison to saline (AOV: *p* < 0.05, BC: *p* < 0.05). An assessment of overall changes in the amount of wake, NREM sleep, and REM sleep revealed no differences between saline and either of the three drugs tested at baseline or post-injection (Baseline: Wake, AOV: F(3,13) = 0.10, *p* = 0.96; NREM, AOV: F(3,13) = 0.25, *p* = 0.86; REM, AOV: F(3,13) = 0.11, *p* = 0.96; Post-injection: Wake, AOV: F(3,13) = 1.34, *p* = 0.30; NREM, AOV: F(3,13) = 1.15, *p* = 0.37; REM, AOV: F(3,13) = 0.66, *p* = 0.59; Fig. 1D). There were also no differences in sleep onset latency (Baseline: AOV: F(3,13) = 0.94, *p* = 0.45; Post-injection: AOV: F(3,13) = 1.45, *p* = 0.27).

To relate these effects on spectral power to the hallmark neurophysiology of sleep, we next quantified drug effects on the spatiotemporal dynamics of automatically detected slow-wave, spindle, and ripple events, based on LFP recordings (Fig. 1E/F). Within the prelimbic cortex (PrL), zolpidem increased the amplitude of slow-waves by 23.7 ± 7.6% and decreased slow-wave intrinsic frequency by 6.4 ± 1.0%, in comparison to saline (Fig. 1G; SW-amplitude, AOV: F(3,13) = 13.86, *p* < 0.001; BC: *p* < 0.001; SW-frequency, AOV: F(3,13) = 2.69, *p* = 0.031; BC: *p* = 0.010). Diazepam decreased the amplitude of slow-waves by 28.0 ± 6.8%, whilst THIP increased the amplitude by 14.6 ± 1.4% in comparison to saline (Fig. 1G; BC: *p* = 0.042 and *p* = 0.040, respectively). None of the drugs altered either the density or the duration of slow-waves in the PrL (Fig. 1G; AOV: F(3,13) = 1.20, *p* = 0.33; F(3,13) = 1.99, *p* = 0.095 respectively). Furthermore, none of the drugs significantly modified sleep spindle density (AOV: F(3,13) = 0.45, *p* = 0.86), amplitude (AOV: F(3,13) = 0.23, *p* = 0.97), intrinsic frequency (AOV: F(3,13) = 0.10, *p* = 1.00), or duration (AOV: F(3,13) = 1.00, *p* = 0.45; Fig. 1H).

Within the dorsal hippocampus CA1, zolpidem increased NREM ripple density compared to baseline, but this effect was not significantly different to saline (Fig. 1I; AOV: F(3,13) = 4.01, *p* = 0.004; BC: *p* = 0.30). Zolpidem did not affect ripple amplitude (AOV: F(3,13) = 28.91, *p* < 0001; BC: *p* = 1.00), intrinsic frequency (AOV: F(3,13) = 13.04, *p* < 0.001; BC: *p* = 0.76), or duration (AOV: F(3,13) = 2.33, *p* = 0.05). In contrast, diazepam markedly decreased ripple amplitude by 56.7 ± 3.8% and increased intra-ripple frequency by 5.0 ± 0.8% in comparison to saline (Fig. 1I; BC: *p* < 0.001; BC: *p* < 0.001, respectively). Diazepam had no effect on ripple density (BC: *p* = 1.00) or duration (BC: *p* = 1.00). THIP had no effect on any ripple features tested (BC: *p* > 0.05).

Slow-waves have previously been shown to bias the timing of spindles, and the degree of this slow-wave spindle coupling impacts memory processing [10, 16]. To identify whether GABA_A_R drug administration alters this temporal relationship, we next measured phase-amplitude coupling (PAC) within the prelimbic cortex (Fig. 2A). PAC analysis of pre to post zolpidem administration revealed a significant increase in coupling between 0.5–1.5 Hz and 10–16 Hz indicating an increase in coupling between slow-waves and spindles (Fig. 2B). This change was significantly different to baseline (CH_zolpidem_ = 47.09 ± 9.40, *t* = 5.01, *p* = 0.007, one-sample *t*-test) and significantly different to saline (*t* = −3.66, *p* = 0.014, two-sampled *t*-test; Fig. 2C). Neither diazepam nor THIP had significant effects on PAC in comparison to saline (diazepam: *t* = −0.69, *p* = 0.535; THIP: *t* = 0.03, *p* = 0.976) or baseline (diazepam: *t* = −1.28, *p* = 0.289; THIP: *t* = −0.36, *p* = 0.743; saline: *t* = −2.78, *p* = 0.069).

### Divergent drug effects on pyramidal cell firing rates and bursting

**Fig. 3:**
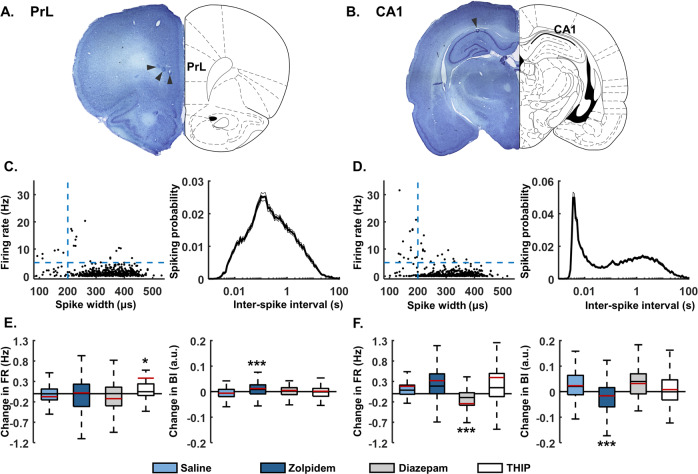
Effects on pyramidal cell firing rates and bursting. Representative micrographs of Thionine-stained 50 µm brain slices showing the sites of post hoc electrolytic lesions (arrowheads) in the prelimbic (PrL) subdivision of the medial prefrontal cortex (**A**) and in the principal cell layer of the dorsal CA1 (**B**) fitted to a schematic of corresponding rat brain section (from the rat brain atlas by Paxinos and Watson, 2007). **C** and **D** left: representative classification of all single units based on average spike widths (µs) and firing rate (FR, Hz) in the PrL (**C**, *n* = 523) and the dorsal CA1 (**D**, *n* = 579). Blue dash lines represent the limits of inclusion for pyramidal cells (mean FR < 5 Hz and spike width > 200 us). **C** and **D** right: mean ± S.E.M interspike interval distribution of PrL pyramidal cells (**C**, *n* = 442) and CA1 pyramidal cells (**D**, *n* = 455). Change in firing rate (left) and bursting index (right) of PrL (**E**) and CA1 (**F**) units after drug injection (40–60 min post injection in comparison to 20 min baseline). Boxplots show the median (black line) and the mean (red line) change in firing rates and bursting index across individual units from all rats. The bottom and top edges of the box indicate the 25th and 75th percentiles, respectively. The whiskers extend to the most extreme data points not considered outliers (for illustrative purposes, outliers have been excluded from the plots). Number of PrL pyramidal cells: saline: *n* = 79, diazepam 4 mg/kg: *n* = 102, zolpidem 3 mg/kg: *n* = 132, THIP 4 mg/kg: *n* = 91; CA1 pyramidal cells: saline: *n* = 85, diazepam 4 mg/kg: *n* = 87, zolpidem 3 mg/kg: *n* = 128, THIP 4 mg/kg: *n* = 83. *, *p* < 0.05 and ***, *p* < 0.001 in comparison to saline, Kruskal–Wallis test followed by Dunns–Sivac post-hoc test. A.u. = arbitrary units.

We next tested how zolpidem, diazepam, and THIP influenced cellular activity within the PrL and the CA1 region of the hippocampus. In the same recordings used for LFP analyses above, we analysed extracellular single-unit activity within these two regions (Fig. 3A/B). A total of 523 units including 475 putative pyramidal cells were identified from the PrL and 579 units including 505 putative pyramidal cells were identified from CA1 (Fig. 3C/D). Changes in firing rate and bursting index were calculated by subtracting the baseline values from the post-drug values. Zolpidem had no effect on PrL or CA1 cell firing rates (PrL: KW: X^2^(3,400) = 11.78, *p* = 0.01, DS: *p* = 1.00; CA1: KW: X^2^(3,379) = 55.2, *p* < 0.001, DS: *p* = 0.73). Diazepam decreased CA1 pyramidal cell firing rates by 0.24 ± 0.01 Hz in comparison to saline (DS: *p* < 0.001; Fig. 3F, left panel). THIP increased PrL pyramidal cell firing rates by 0.39 ± 0.12 Hz in comparison to saline (DS: *p* = 0.049; Fig. 3E, left panel).

Alongside influencing firing rates, GABAergic signaling also regulates burst firing [67]. We therefore quantified the effects of the different drugs on pyramidal cell burst-firing activity (ISI < 20 ms, bursting index, BI). Unexpectedly, zolpidem had opposite effects in the two regions, increasing the bursting activity of PrL pyramidal cells (change in BI = 0.01 ± 0.00; KW: X^2^(3,400) = 15.90, *p* = 0.001, DS: *p* < 0.001; number of cells: saline = 79, zolpidem = 132; Fig. 3E, right panel) while decreasing bursting in CA1 cells (change in BI = −0.02 ± 0.01; KW: X^2^(3,379) = 40.17, *p* < 0.001, DS: *p* < 0.001; ncells = 85/128; Fig. 3F, right panel). THIP and diazepam had no effect on bursting activity (DS: *p* > 0.05).

### Zolpidem enhances slow-wave modulation of pyramidal cells in PrL and CA1

**Fig. 4:**
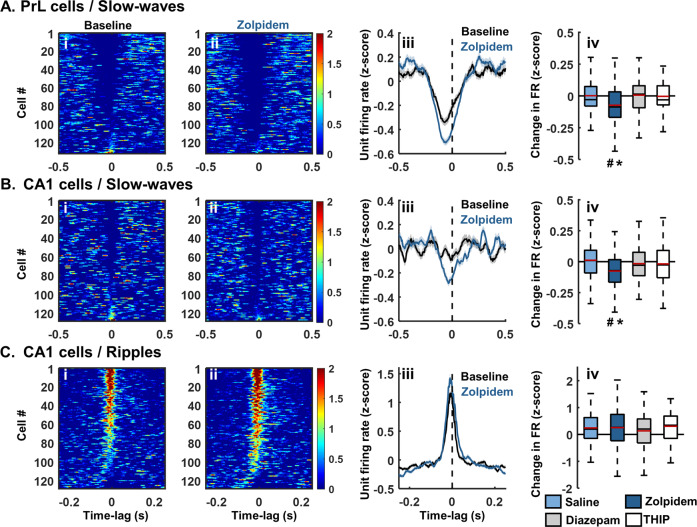
Modulation of cell activity. **A**, **B** peri-event time histograms (PETH) showing alterations of firing rates of PrL units or CA1 units during slow-wave oscillations following GABAAR modulator injections (±500 ms centred on slow-wave negative peak, time = 0; comparison 20 min baseline to 40–60 min post drug, 5 ms bins). **C** PETH showing alterations of firing rates of CA1 units during ripples following GABAAR modulator injections (±250 ms centred on ripples maxima, time = 0; comparison 20 min baseline to 40–60 min post drug, 2 ms bins). **A**-**C** from left to right, normalised PETH for all units before (i) and after (ii) 3 mg/kg zolpidem injection. (iii) population average PETH (mean ± S.E.M, z-score, individual units from all rats) before and after zolpidem 3 mg/kg injection. (iv) Boxplots show the median (black line) and the mean (red line) change in firing rate between pre and post drug across individual units from all rats (**A** and **B**, calculated between −250 ms and +250 ms around the slow-wave negative peak; **C** calculated between −40 ms and +40 ms around the ripple maxima). The bottom and top edges of the box indicate the 25th and 75th percentiles, respectively. The whiskers extend to the most extreme data points not considered outliers (for illustrative purposes, outliers have been excluded from the plots). Number of PrL pyramidal cells: saline: *n* = 79, diazepam: *n* = 102, zolpidem: *n* = 132, THIP: *n* = 91; CA1 pyramidal cells: saline: *n* = 85, diazepam: *n* = 87, zolpidem: *n* = 128, THIP: *n* = 83. Asterisks denote statistical significance in comparison to saline injection: *p < 0.05, hashes represent statistical differences to baseline: #, *p* < 0.05, one-way ANOVA followed by Bonferroni post-hoc test.

Slow-waves are signatures of cortical network transitions between high and low activity states; a depolarized UP state associated with neuronal spiking and a low-activity DOWN state with little firing or synaptic drive [68]. This firing rate modulation is evident in peri-event histograms of PrL cell firing centered on the negative peaks of slow-waves (Fig. 4Ai, example of baseline recording before zolpidem injection, number of cells = 132). Zolpidem accentuated the effect of the DOWN state on PrL pyramidal cells: cell firing rates showed a greater reduction at the peak of the slow-wave after zolpidem injection compared to baseline (calculated 250 ms around the peak of SW, change in FR_zscore_ = −0.07 ± 0.01; AOV: F(3,423) = 6.85, *p* < 0.001, BC: *p* = 0.002 in comparison to saline; ncells: saline = 79, zolpidem = 132; Fig. 4Aii/iii). This effect of zolpidem was also evident in CA1 (change in FR_zscore_ = −0.08 ± 0.01; AOV: F(3,400) = 6.12, *p* < 0.001, BC: *p* = 0.001 in comparison to saline; ncells = 85/128; Fig. 4Bii/iii). However, zolpidem did not significantly modify CA1 cell firing rates during ripples in comparison to saline (AOV: F(3,379) = 0.92, *p* = 0.43; Fig. 4C). Neither diazepam nor THIP changed the modulation of PrL or CA1 cell firing rates by slow-waves, or CA1 cell firing rates by ripples (Fig. 4iv).

### Zolpidem enhances cross-correlated cell pair activity spanning CA1 and PrL

**Fig. 5:**
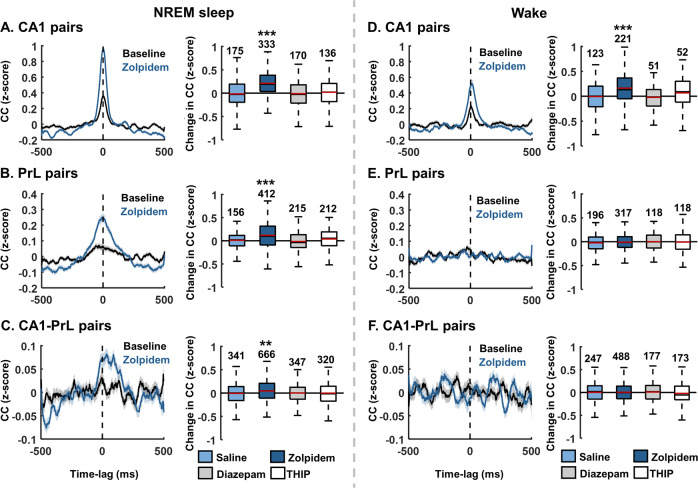
Cell pair cross correlations. Normalised cross-correlograms between CA1 units (**A**), PrL units (**B**) and PrL–CA1 units (**C**) during NREM sleep or wake (**D**–**F**) following GABAAR modulator injections (±500 ms centred on the spiking time of the first cell of the pair, time = 0; comparison 20 min baseline to 40-60 min post drug, 5 ms bins). Left panels, cross-correlation (z-score, mean±S.E.M, across individual unit pairs from all rats) before and after 3 mg/kg zolpidem injection (20 min baseline vs 40–60 min post-drug injection). Right panels, box plots show the median (black line) and the mean (red line) change in peak cross-correlation after drug injection across individual units from all rats, calculated between −100 and +100 ms. The bottom and top edges of the box indicate the 25th and 75th percentiles, respectively. The whiskers extend to the most extreme data points not considered outliers (for illustrative purposes, outliers have been excluded from the plots). The number of cell pairs are shown above the whiskers. *, *p* < 0.05, **, *p* < 0.01 and ***, *p* < 0.001, comparison to saline, Kruskal–Wallis test followed by Dunns-Sivac post-hoc test. (**D**–**F**), same, but during wake periods.

Finally, we assessed the pyramidal cells’ capacity to fire in synchrony (reflecting neural ensembles) and its alteration by the different GABAARs modulators. We measured cross-correlations between all the putative pairs of cells in the CA1, PrL and between PrL and CA1 Overall, only zolpidem altered intra-regional cross-correlations, significantly increasing the levels of cross-correlation in both CA1 (KW: X^2^(3,769) = 112.24, *p* < 0.001, DS: *p* < 0.001; number of cells: saline = 171, zolpidem = 293) and PrL (KW: X^2^(3,1073) = 27.79, *p* < 0.001, DS: *p* < 0.001; n cells = 229/341) in comparison to saline. Similarly, only zolpidem modified the inter-region cross-correlation causing an increase between the CA1 and PrL (KW: X^2^(3,1667) = 20.13, *p* < 0.001, DS: *p* = 0.027; ncells = 365/641). To discern possible differential effects between zolpidem’s action during NREM sleep and wake we further calculated cross-correlations separately for these vigilance states. As shown in Fig. 5A–C, only zolpidem altered intra-regional and inter-regional cross-correlations specifically during NREM sleep (CA1-CA1, KW: X^2^(3,810) = 93.06, *p* < 0.001, DS: *p* < 0.001; PrL-PrL, KW: X^2^(3,991) = 40.15, *p* < 0.001, DS: *p* < 0.001; CA1-PrL, KW: X^2^(3,1670) = 19.22, *p* < 0.001, DS: *p* = 0.007). During wake, zolpidem again augmented inter-regional cross-correlations within CA1 (CA1-CA1, KW: X^2^(3,443) = 26.43, *p* < 0.001, DS: *p* < 0.001; Fig. 5D). However, this effect was completely absent within the PrL (PrL-PrL, KW: X^2^(3,745) = 0.88, *p* = 0.830; Fig. 5E) and between the CA1 and PrL (CA1-PrL, KW: X^2^(3,1081) = 2.53, *p* = 0.469; Fig. 5F). All significant effects found comparing zolpidem to saline were also confirmed between zolpidem pre-injection baseline and post-injection.

## Discussion

We identified differential effects of the GABA_A_R modulators zolpidem, diazepam, and THIP on oscillatory activity during NREM sleep in adult rats. At the network level, we confirm that zolpidem increased cortical slow-wave and hippocampal ripple activity, plus demonstrate that it enhanced coupling between slow-waves and spindles. At the cellular level, zolpidem augmented slow-wave-modulation of firing rates in both cortex and hippocampus and, of particular note, significantly increased coordinated firing within and between the hippocampus and prefrontal cortex. In contrast, whilst diazepam led to a decrease in slow-wave and ripple activity and THIP led to an increase in slow-wave activity, neither drug systematically altered cell firing rates or synchronous activity.

### The pharmacology of hypnotic drug effects on NREM sleep oscillations

Zolpidem’s sedative effects are mediated primarily by α1GABA_A_Rs [69], hence mice carrying zolpidem-insensitive α1GABA_A_Rs do not present changes in sleep EEG after 5 mg/kg systemic zolpidem [52]. This suggests that both sedative and oscillatory effects are mediated by the activation of α1 subunits, though high doses of zolpidem can also modulate α2GABA_A_Rs and decrease SWA [52, 53, 70]. In our study, 3 mg/kg zolpidem increased the amplitude of slow-waves in PrL, corroborating similar increases in slow-wave power reported in previous rodent studies [55, 56, 59, 71] and in humans [72, 73]. A moderate dose of zolpidem is, therefore, more likely to be selective for α1 subunits, and to enhance—rather than disrupt—coordinated activity during NREM sleep. In contrast, the reduction of SWA by diazepam is typical of benzodiazepines in both humans [41, 51] and rodents [43, 46, 59]. Like zolpidem, diazepam’s sedative effects are linked to α1GABAAR activation [69], but its effects on SWA are also dependent on the activation of α2GABAARs, since mice expressing a mutated version of α2 do not show a decrease in SWA after diazepam injection [57].

Pyramidal cell α1GABAARs are mainly activated by GABA released by parvalbumin-expressing interneurons, inducing phasic inhibition [74]. Zolpidem increases the amplitude and duration of inhibitory post-synaptic potentials onto pyramidal cells [75], and a longer and stronger inhibitory input could contribute to the decrease in frequency and increase in amplitude of slow-wave oscillations observed in this study. Furthermore, we observed an increase in the PrL neuronal bursting activity during zolpidem treatment, which could also fuel the increase in slow-wave amplitude.

THIP also increased slow-wave amplitude, consistent with studies in both humans [50] and rodents [54, 61], most likely through mechanisms dissociable from those targeted by zolpidem. δGABA_A_Rs are expressed extrasynaptically in the thalamus, where they induce tonic inhibition of thalamocortical neurons in vitro and promote burst firing patterns consistent with the firing patterns observed during NREM sleep [76]. THIP could also act locally by increasing the tonic inhibition of local interneurons [77], which could contribute to the increase in PrL firing rates induced by THIP observed in our study.

In our study, no drugs altered spindle amplitudes or densities whereas, in humans, benzodiazepines and Z-drugs can augment spindle power and density [11, 41, 50, 51], and THIP decreases spindle activity [50, 78]. These differences may reflect dose and metabolism, or interactions between drug effects and memory processing in human studies—for example, drugs may augment learning-dependent increases in spindle density. This could be tested in rodents by using cognitive training to probe interactions between zolpidem and experience-dependent spindle activity.

### Zolpidem promotes ripples and coordinated activity in the CA1

Zolpidem increased ripple density in the CA1 region of the hippocampus. This result contradicts the decrease in ripple occurrence, amplitude, and frequency induced by 10 mg/kg zolpidem in a previous study [47]. However, in vitro, low doses of zolpidem can increase ripple power, while high doses induce the opposite effect, suggesting a U-shape dose-dependence and subunit-selective effect of the drug [48].

We also observed an increase in overall synchrony between the CA1 pyramidal cell pairs induced by zolpidem. This may reflect α1GABA_A_R activation increasing the amplitude of IPSPs onto pyramidal cells [79, 80] and amplifying the phasic inhibition known to synchronize populations of pyramidal cells [81]. We also show that zolpidem enhanced entrainment of CA1 pyramidal cells by slow oscillations recorded in the PrL, an effect thought to be mediated via the entorhinal cortex [82]. We speculate that zolpidem amplifies input from the entorhinal cortex to the hippocampus (via slow-wave synchrony), promoting sharp wave-ripples and synchronization between pyramidal cells in CA1.

Diazepam markedly decreased ripple amplitude in the CA1, which is consistent with previous reports in vivo [47, 83] and in vitro [48]. The diazepam-induced increase in intrinsic ripple frequency is surprising as it contradicts the effects of diazepam described by others (either decrease: [47, 48] or no effect: [84]). However, diazepam did increase the overall bursting activity of CA1 pyramidal cells which may contribute to the increase in intrinsic ripple frequency observed here [85].

We did not observe any effect of THIP on ripple oscillations or CA1 cell firing during ripples, suggesting a lack of involvement of tonic δGABA_A_RS in the regulation of ripples.

### Zolpidem enhances hippocampal-prefrontal coupling during NREM sleep: implications for NREM sleep-dependent memory consolidation

Our principal hypothesis was that zolpidem’s beneficial effects on sleep-dependent memory consolidation [8–11] would reflect modulation of limbic-cortical interactions during non-REM sleep. While zolpidem did not induce significant changes to spindle features, it did significantly increase phase-amplitude coupling between spindles and slow-waves, alongside enhancing slow-wave modulation of CA1 activity and correlation of CA1-PrL spiking. Zolpidem does therefore appear to promote coordinated CA1-PrL activity in a manner consistently shown to benefit memory processing in both rodents and humans [24–26]—though we did not test behavioral effects in this study and stronger inferences could be drawn from simultaneous monitoring of zolpidem’s effects on neurophysiology and behavior. This is in contrast to diazepam, which suppressed both slow-wave and ripple activity, and can have detrimental effects on memory consolidation [86, 87].

Niknazar et al. (2015) [10] previously identified both a significant increase in spindle density and phase-amplitude coupling between spindles and slow-waves following zolpidem in humans. This may reflect increased inhibition from the thalamus, which has been predicted to increase network synchronisation [88] and is central to a model of coordinated limbic, thalamic, and cortical interactions supporting NREM-dependent information processing [17]. In this model, hippocampal-cortical dialogue during NREM is tuned by the relative timing of slow-waves, spindles, and ripples, hence augmenting their coordination can enhance memory [24, 28]. We provide cellular-resolution evidence for zolpidem’s net enhancement of limbic-cortical interactions, framing future experiments designed to quantify drug effects on encoding, reactivation, and storage of specific types of salient information during learning and memory. It should be noted, however, that zolpidem does impair cognition tested during awakenings shortly after administration [89, 90], hence the relative timing of learning, testing and dosing—and effects on memories at different timepoints after learning—should be further characterized.

## Conclusions

In conclusion, this study reveals novel insights into the differential effects of GABA_A_ receptor modulators on underlying neural activity during sleep. Compared with diazepam and THIP, our results suggest that zolpidem is most effective at enhancing natural sleep oscillatory activity and cortico-limbic synchronisation through a modification of underlying cellular activity. However, the timing of zolpidem relative to sleep onset and circadian phase, and the dose-dependence of its potential to enhance limbic-cortical interactions, bear further exploration. For instance, the typical clinical dose of 10 mg zolpidem shortly before sleep onset in humans has not explicitly been optimized in terms of mnemonic or neurophysiological metrics, hence different doses or timings may better support memory consolidation. Future studies using a range of dosages, longer-term treatments, and a direct assessment on memory, particularly in relation to hippocampal replay, will further advance this understanding. Zolpidem could therefore represent a drug of choice in treatment for sleep disorders that would ensure maintenance or even enhancement of memory consolidation, given the correct dosage and potential combination with digital health or talking therapies. It will also be important to continue quantifying zolpidem’s effects on NREM oscillations in diagnoses associated with disordered slow-waves, spindles and/or slow-wave spindle coupling, including ADHD [91], schizophrenia [34, 35], and Alzheimer’s disease [37].

## Supplementary information

Table 1
