# Supplementary material for: The GABAA receptor modulator zolpidem augments hippocampal-prefrontal coupling during non-REM sleep
Source: Neuropsychopharmacology. 2022 Jun 18;48(4):594–604. doi: 10.1038/s41386-022-01355-9 (PMC9938179; doi:10.1038/s41386-022-01355-9)
Supplement: Supplementary file 1 — Table 1 [file 41386_2022_1355_MOESM1_ESM.docx]

| **Characteristic** | **Drug** | **Pre-injection baseline** | **Post-injection** |
| --- | --- | --- | --- |
| **Slow-waves:** |  |  |  |
| Density (s^-1^) | Saline | 0.070±0.014 | 0.079±0.009 |
|  | Zolpidem | 0.074±0.011 | 0.115±0.028 |
|  | Diazepam | 0.072±0.015 | 0.109±0.031 |
|  | THIP | 0.086±0.008 | 0.115±0.030 |
| Amplitude (μV) | Saline | 540.15±78.56 | 501.16±72.87 |
|  | Zolpidem | 518.17±81.72 | 629.85±79.40 |
|  | Diazepam | 368.88±54.13 | 275.14±63.43 |
|  | THIP | 464.10±52.18 | 532.23±62.69 |
| Frequency (Hz) | Saline | 2.44±0.17 | 2.52±0.15 |
|  | Zolpidem | 2.49±0.12 | 2.33±0.12 |
|  | Diazepam | 2.54±0.14 | 2.50±0.09 |
|  | THIP | 2.43±0.08 | 2.40±0.15 |
| Duration (s) | Saline | 0.58±0.02 | 0.57±0.02 |
|  | Zolpidem | 0.63±0.05 | 0.70±0.05 |
|  | Diazepam | 0.59±0.04 | 0.55±0.04 |
|  | THIP | 0.56±0.03 | 0.58±0.03 |
| **Spindles:** |  |  |  |
| Density (s^-1^) | Saline | 0.059±0.010 | 0.069±0.007 |
|  | Zolpidem | 0.063±0.002 | 0.073±0.010 |
|  | Diazepam | 0.058±0.003 | 0.071±0.012 |
|  | THIP | 0.055±0.009 | 0.068±0.016 |
| Amplitude (μV) | Saline | 378.90±76.98 | 375.60±80.30 |
|  | Zolpidem | 404.27±87.89 | 393.97±90.75 |
|  | Diazepam | 309.77±126.71 | 297.43±109.72 |
|  | THIP | 321.37±78.32 | 323.85±83.40 |
| Frequency (Hz) | Saline | 12.03±0.19 | 12.00±0.11 |
|  | Zolpidem | 12.03±0.05 | 12.03±0.07 |
|  | Diazepam | 12.11±0.10 | 12.02±0.21 |
|  | THIP | 12.11±0.14 | 12.04±0.12 |
| Duration (s) | Saline | 1.02±0.08 | 0.94±0.08 |
|  | Zolpidem | 1.04±0.12 | 0.94±0.11 |
|  | Diazepam | 1.06±0.14 | 0.98±0.13 |
|  | THIP | 0.90±0.04 | 0.87±0.04 |
| **Ripples:** |  |  |  |
| Density (s^-1^) | Saline | 0.220±0.017 | 0.248±0.003 |
|  | Zolpidem | 0.241±0.025 | 0.358±0.052 |
|  | Diazepam | 0.244±0.015 | 0.318±0.052 |
|  | THIP | 0.222±0.037 | 0.281±0.072 |
| Amplitude (μV) | Saline | 387.47±48.43 | 395.48±37.75 |
|  | Zolpidem | 348.05±22.06 | 375.89±16.25 |
|  | Diazepam | 374.00±67.59 | 158.47±27.72 |
|  | THIP | 399.60±47.61 | 377.50±40.46 |
| Frequency (Hz) | Saline | 155.90±0.73 | 156.35±0.76 |
|  | Zolpidem | 158.81±1.39 | 156.49±1.69 |
|  | Diazepam | 156.60±1.34 | 164.46±0.81 |
|  | THIP | 157.60±0.59 | 156.11±1.17 |
| Duration (s) | Saline | 0.072±0.003 | 0.071±0.003 |
|  | Zolpidem | 0.071±0.003 | 0.073±0.003 |
|  | Diazepam | 0.071±0.001 | 0.065±0.001 |
|  | THIP | 0.074±0.002 | 0.071±0.002 |

**The GABA_A_ receptor modulator zolpidem augments hippocampal-prefrontal coupling during non-REM sleep**

Flavie Kersanté Ph.D.^1^, Ross J. Purple Ph.D.^1^ and Matthew W. Jones Ph.D.*

**Supplementary results**

Table 1: Absolute values for slow-wave, spindle, and ripple characteristics during the 20-minute analysis windows pre- and post-injection (mean±SEM).
